# Reorganization of wheat and rye genomes in octoploid triticale (× *Triticosecale*)

**DOI:** 10.1007/s00425-017-2827-0

**Published:** 2017-12-12

**Authors:** Anna Kalinka, Magdalena Achrem

**Affiliations:** 1 Department of Cell Biology, Faculty of Biology, Institute for Research on Biodiversity, University of Szczecin, Wąska 13, 71-415 Szczecin, Poland; 2 Faculty of Biology, Molecular Biology and Biotechnology Center, University of Szczecin, Wąska 13, 71-415 Szczecin, Poland

**Keywords:** Allopolyploids, Chromosome elimination, Genomic changes, Retrotransposons, Sequence rearrangement

## Abstract

**The analysis of early generations of triticale showed numerous rearrangements of the genome. Complexed transformation included loss of chromosomes, t-heterochromatin content changes and the emergence of retrotransposons in new locations.**

This study investigated certain aspects of genomic transformations in the early generations (F5 and F8) of the primary octoploid triticale derived from the cross of hexaploid wheat with the diploid rye. Most of the plants tested were hypoploid; among eliminated chromosomes were rye chromosomes 4R and 5R and variable number of wheat chromosomes. Wheat chromosomes were eliminated to a higher extent. The lower content of telomeric heterochromatin was also found in rye chromosomes in comparison with parental rye. Studying the location of selected retrotransposons from *Ty1*-*copia* and *Ty3*-*gypsy* families using fluorescence in situ hybridization revealed additional locations of these retrotransposons that were not present in chromosomes of parental species. ISSR, IRAP and REMAP analyses showed significant changes at the level of specific DNA nucleotide sequences. In most cases, the disappearance of certain types of bands was observed, less frequently new types of bands appeared, not present in parental species. This demonstrates the scale of genome rearrangement and, above all, the elimination of wheat and rye sequences, largely due to the reduction of chromosome number. With regard to the proportion of wheat to rye genome, the rye genome was more affected by the changes, thus this study was focused more on the rye genome. Observations suggest that genome reorganization is not finished in the F5 generation but is still ongoing in the F8 generation.

## Introduction

Synthetic allopolyploids are most often obtained by interspecific or intergeneric hybridization, followed by chromosome doubling in hybrids. Certain phenomena that accompany the naturally occurring allopolyploidization could be distinguished thanks to the possibility of creating such synthetic allopolyploids. The diploidization processes proceed differently and have a different nature in individual allopolyploid species (Liu and Wendel 2002). The genomic response to allopolyploidization is therefore species-specific. In several species, there are virtually no changes and parental genomes remain unchanged. However, for most allopolyploids, large-scale modifications of the emerging hybrid genome have been demonstrated. These transformations often occur in a relatively short period of time, immediately after the hybridization and/or polyploidization (Teutonico and Osborn 1994; Wendel et al. 1995).

Allopolyploidization enhances the ability of plants to adapt to environmental conditions; however, the multiplication of genes and genomes often causes genomic instability, chromosome number imbalance, regulatory incompatibility and decreased fertility. Foreign genomes in hybrids are subject to specific transformations that must occur in order to adapt them to harmonious interaction (Comai 2000; Liu and Wendel 2002; Ma and Gustafson 2005).

Analysis of the genome of some allopolyploid species has shown that parental genomes in the hybrid are being restructured (Comai 2000; Ma and Gustafson 2005; Udall et al. 2005), and for this reason genomes of stabilized allopolyploids differ from parental genomes (Bennett et al. 1992; Kotseruba et al. 2003). It has also been noted that the elimination of certain DNA nucleotide sequences is one of the major trends, in addition to changes in chromosome structure or rearrangements of the genomic nucleotide sequence (Liu et al. 1998; Ozkan et al. 2001, 2002; Feldman and Levy 2005). It is suggested that the elimination of nucleotide sequences is the main aspect of the allopolyploidization in many species, treated downright as the driving force of this process. It was proven that some types of sequences were lost from the hybrid genome while the chromosome number remained stable (Comai et al. 2000; Xiong et al. 2011). However, the mechanism responsible for this elimination is still unknown; it is not clear when and how the initiation of sequence elimination occurs (Ozkan et al. 2001, 2003). It seems likely that the role of sequence elimination is to increase homeologous chromosomal heterogeneity in allopolyploids by allowing meiosis to follow the diploid pattern.

Another type of synthetic alloploid reaction to the genomic shock are the epigenetic alterations, which lead to development of new genome regulation (Madlung and Comai 2004). The differently regulated parental genomes have to cooperate when combined in the allopolyploid genome. At the same time, the problem of excessive gene dosage must be resolved. Hence epigenetic changes are particularly important for the stabilization and adaptation of the new allopolyploid species (Madlung et al. 2002), although they have not been observed in all species (Liu et al. 2001). However, most of the newly formed allopolyploids remodeled their epigenetic patterns (Comai 2000; Pikaard 2001; Madlung et al. 2002; Osborn et al. 2003; Feldman and Levy 2005; Lukens et al. 2006; Gaeta et al. 2009). As a consequence, changes in epigenetic regulation in allopolyploids can lead to the mobile genetic elements movement (Comai 2000; Liu and Wendel 2000; Kashkush et al. 2003). The release of the mass transposition of mobile genetic elements can lead to many mutations affecting the chromosome structure and function of the genome (Comai 2000). Furthermore, regulatory changes regarding mobile genetic elements may interfere with the expression of adjacent genes (Kashkush et al. 2003). The influence of mobile genetic elements on the remodeling of the allopolyploidic genome can be very significant, especially given the huge number of their copies in plant genomes.

Only certain aspects of genomic transformations in early generations of wheat–rye allopolyploids have been investigated in this study, but the analysis shows a fairly wide spectrum of changes. This study is unique in this aspect, that the common wheat cultivar Lanca, which is the maternal form of the hybrids investigated in this study, has been subjected to chromosomal engineering (Tarkowski and Apolinarska 1992). It was crossed with the dwarf form of rye, LT 506/79, and then the number of chromosomes was doubled in the F1 hybrid to obtain the octoploid triticale line. Then, this primary triticale was backwardly crossed with wheat and the presence of chromosome pair 1RS.1BL was found after several years of culture. As the Lanca wheat used in this study had a hybridization with rye event in its history (1RS.1BL translocation was confirmed), it was interesting how another hybridization would proceed. The aim of the study was to analyze the rearrangement of DNA nucleotide sequences in hybrid genomes and to determine differences in chromosome number in the studied lines and to determine the changes of location of retrotransposons in chromosomes. The research was conducted in two generations of hybrids to obtain information about their possible genetic stabilization. The results demonstrated the types, directions and intensity of genomic changes occurring in the early generations of wheat–rye allopolyploids.

## Materials and methods

### Plant materials

Hybrids were created by crossing hexaploid wheat (*Triticum aestivum* ssp. *vulgare* L.) cultivar Lanca (Poland) with diploid rye (*Secale cereale* L.) cultivar Muro (Germany). Plants of the F1 generation were treated with colchicine to double the number of chromosomes in the genome. According to the cytological test, F1 plants had 56 chromosomes in somatic cells. Due to the fact that kernels of each plant were sown separately starting from F1, their progeny was designated as lines. Primary octoploidal triticale from Lanca × Muro cross was designated as LMUR. Lines were propagated for several generations by self-pollination. Five lines of F5 and F8 plant generations were analyzed: LMUR 51, 54, 60, 61 and 107. The tested plants in the F8 generation were the progeny of the F5 generation. Cultivars of parental species served as control genotypes. Lanca wheat exhibited the presence of chromosome pair 1RS.1BL. Hybrids were obtained at the Department of Genetics and Plant Breeding, Poznan University of Life Sciences. The seeds of parental species were obtained from the Institute of Plant Breeding and Acclimatization in Radzikow (Radzików, 05-870 Błonie, Poland).

### Determining chromosome number, C band analysis in rye chromosomes

The roots of the 2-day seedlings were treated with 0.05% colchicine solution for 3 h, and then fixed in aceto-alcohol (1:3) at 4 °C for 24 h. The material was macerated in a mixture of 4% (w/v) pectinase (Sigma-Aldrich, St. Louis, MO, USA), 6% (w/v) hemicellulase (Sigma-Aldrich) and 4% (w/v) cellulase (Sigma-Aldrich) in 0.01 M citrate buffer (pH 4.8) for 3 h at 37 °C. The preparations were made in a drop of 45% acetic acid. Giemsa staining was performed according to Darvey’s and Gustafson’s modification (1975). Fifty plants of each triticale line/generation were studied. Five metaphase spreads per each plant were analyzed. Changes in the C-banding pattern in triticale were determined by comparison with the standard, i.e., with patterns defined for the direct offspring of Lanca wheat (1RS.1BL) and Muro rye (1R–7R) parental plants. The preparations were analyzed using an Eclipse E600 microscope (Nikon, Tokyo, Japan). Measurements were carried out using NIS Elements ver. 3.00 SP7 software (Nikon). Based on the measurements, the percentage of telomeric heterochromatin (% th) was determined for individual arms and entire chromosomes. Analyses of the differences in heterochromatin content were performed using the Kruskal–Wallis test (Statistica software ver. 12) with multiple comparisons. The test probability was considered significant at *P* < 0.05 and highly significant at *P* < 0.01.

### Occurrence and location of selected retrotransposons

DNA was isolated from 1 g of freshly collected triticale, rye and wheat coleoptiles using the modified CTAB method (Murray and Thompson 1980). To detect the presence of retrotransposons belonging to the *Ty1*-*copia* and *Ty3*-*gypsy* families, PCR reaction was performed with degenerate primers allowing to obtain a fragment of the reverse transcriptase (RT) gene: for *Ty1*-*copia* (Flavell et al. 1992a, b): 5′ACNGCNTTYYTNCAYGG and 5′ARCATRTCRTCNACRTA, for *Ty3*-*gypsy* (Kubis et al. 1998): 5′MRNATGTGYGTNGAYTAYMG and 5′RCAYTTNSWNARYTTNGCR. These primers were also used to label molecular probes. All primers were synthesized in the IBB Laboratory of DNA Sequencing and Synthesis in Warsaw. The reaction was carried out in a DNAEngine^®^Peltier Thermal Cycler (Bio-Rad, Hercules, CA, USA) thermocycler. Each reaction was performed three times in order to make the results reliable. *Ty1*-*copia* reactions were carried out in a 25-μL reaction mixture which contained: 25 ng DNA, 1 × buffer (Fermentas, Vilnius, Lithuania), 2 mM MgCl_2_, 0.16 mM dNTP (Fermentas), 0.8 μM each primer, 1 U polymerase (DreamTaq™, Fermentas). The 25 μL *Ty3*-*gypsy* reaction mixture contained: 25 ng DNA, 1 × buffer (Fermentas), 2 mM MgCl_2_, 0.16 mM dNTP (Fermentas), 0.8 μM each primer, 0.75 U polymerase (DreamTaq™, Fermentas). The thermal profile included the following PCR reaction steps: (1) initial DNA denaturation: 95 °C for 5 min; (2) 30 cycles: DNA denaturation at 94 °C for 50 s, primer annealing at 39–42 °C for 50–60 s, DNA chain elongation at 72 °C for 40–60 s; (3) final extension at 72 °C for 7 min. Amplification products were separated in 1.5% agarose gel in 1 × TBE for 4 h at 80 V. Ethidium bromide (Sigma-Aldrich) was added to the gel at a concentration of 0.1 μg mL^−1^. Amplification product sizes were estimated by comparison with the standard mass marker—MassRuler™ DNA Ladder Mix (Fermentas). Electrophoresis was carried out in a Sub-cell^®^ Model 192 (Bio-Rad) connected to a PowerPacTM Basic power supply (Bio-Rad). The results were recorded using Gel Doc XR (Bio-Rad) in the Quantity One^®^ (Bio-Rad) program, which was used for electropherogram analyses.

Fluorescence in situ hybridization (FISH) preparations were incubated in RNase (DNase-free, 10 μg mL^−1^) (Sigma-Aldrich) in 2 × SSC buffer (0.03 M sodium citrate, 0.3 M NaCl, pH 7.0) for 1 h at 37 °C. Then they were washed twice with 2 × SSC buffer for 5 min at 37 °C and incubated in 0.12% pepsin solution (Carl Roth GmbH, Karlsruhe, Germany) for 15 min at 37 °C. Samples were re-washed twice with 2 × SSC buffer for 5 min at 37 °C, dehydrated in an ethanol series (70, 90 and 99.8%) at RT. For retrotransposon localization, probes of the reverse transcriptase gene fragments of retrotransposons from *Ty1*-*copia* and *Ty3*-*gypsy* families were prepared. The labeling was performed by PCR in a 50-µL reaction mixture containing 50 ng of total DNA, 1 mM of each oligonucleotide primer, 0.2 mM dATP, dCTP, 0.04 mM biotin-11-dUTP (Thermo Scientific, Vilnius, Lithuania), 1 × buffer, 2.5 mM MgCl_2_ and 1–1.5 U Taq DNA polymerase (AllegroTaq, Novazym, Poznan, Poland). Thermal conditions of the PCR reaction were identical as in the amplification of *Ty1*-*copia* and *Ty*-*3 gypsy* reverse transcriptase gene. The labeled molecular probe was purified using the Gene-Matrix PCR/DNA Clean-Up Purification Kit (EUR_x_, Gdansk, Poland). The concentration of the probe was determined spectrophotometrically (SmartSpec™ Plus, Bio-Rad). The probes were mixed with a hybridization mixture containing: 45% formamide, 9% dextran sulfate (Sigma-Aldrich), 0.9% Tween 20, 1.8 × SSC buffer, 100 ng μL^−1^ salmon sperm DNA (Roche Diagnostics, Indianapolis, IN, USA). The final concentration of each probe was 1 ng μL^−1^. Denaturing the probe and chromosomal DNA was run for 5 min at 83 °C. Hybridization was carried out for 16 h at 37 °C. Post-hybridization wash was carried out with 30% formamide and 2 × SSC (3 × 5 min) and 2 × SSC buffer alone (3 × 5 min) at 43 °C. Preparations were incubated in DB (detection buffer) (4 × SSC, 0.2% Tween 20) at 37 °C for 5 min. Probe detection was performed using avidin-FITC (5 μg mL^−1^) (Sigma-Aldrich). Signal amplification was performed by incubation in an anti-avidin antibody (5 mL) (Vector Laboratories, Burlingame, CA, USA) followed by incubation in avidin-FITC (5 μg mL^−1^) (Sigma-Aldrich). Signal amplification was repeated twice and the preparations were washed 3 times in DB buffer for 5 min at 37 °C after each incubation. After preparation dehydration, chromosomes were stained with DAPI reagent (1 μg mL^−1^) (Sigma-Aldrich) for 15 min. The preparations were analyzed using an Eclipse E600 epifluorescence microscope (Nikon). Images were recorded and analyzed in Genus software (Applied Imaging, San Jose, CA, USA).

### Sequence rearrangement analysis: ISSR, IRAP and REMAP

For the amplification of inter-microsatellite sequences, 47 arbitrary 3′-anchored primers were designed, of which 20 selected primers were used for the analysis (Table 1). Eight primers were designed for the IRAP method, of which 4 were selected for the analysis (Table 1). The REMAP method used primer combinations of microsatellite sequences and retrotransposons sequences (Table 1). Of the 50 combinations tested, 16 were selected. FastPCR was used to design primers for retrotransposon sequences (Kalendar et al. 2009). *Angela* retrotransposon primers were designed using sequences from the NCBI database with AY485644 accession number, for *Cassandra*—AY359471, for *Bilby*—AF245032 and for *Sukkula*—AY223546. Retrotransposon sequences are derived from cereal genomes of the genus *Secale* (*Cassandra*, *Sukkula, Bilby*) or *Triticum* (*Angela*). All primers were synthesized in the IBB Laboratory of DNA Sequencing and Synthesis in Warsaw. ISSR, REMAP and IRAP reactions were carried out in a 20-μL reaction mixture that contained, depending on the primer: 50–400 ng DNA, 1 × buffer (Novazym), 4.4–7.5 mM MgCl_2_, 0.2–0.4 mM dNTP (Fermentas), 1–2 μM primer, 1.25–5 U polymerase (RED AllegroTaq, Novazym). The composition of the reaction mixture and the thermal profile of the reaction were experimentally determined. The thermal profile included: (1) denaturation: 95 °C for 5 min; (2) 35 cycles: denaturation at 94 °C for 40 s, annealing at 37–60 °C for 60 s, elongation at 72 °C for 120 s; (3) final extension at 72 °C for 5 min. The thermal profile was the same for all reactions except for primer annealing temperature (Table 1). The reaction was carried out in a DNAEngine^®^Peltier Thermal Cycler (Bio-Rad). Each reaction was performed in triplicate on separately isolated DNA samples in order to make the results reliable. Amplification products were separated in 2% agarose gel in 1 × TBE with ethidium bromide at a concentration of 0.1 μg mL^−1^. Electrophoresis was performed in 1 × TBE buffer at 80 V. Amplification product sizes were estimated by comparison with a standard mass marker (MassRuler™ DNA Ladder Mix, Fermentas). Electrophoresis was performed on a Sub-cell^®^ Model 192 apparatus (Bio-Rad). The results of the electrophoresis were recorded using Gel Doc XR (Bio-Rad) in the Quantity One^®^ (Bio-Rad) program, which was used for electropherogram analyses. The obtained data were used to generate a similarity matrix and dendrogram using FreeTree software (Pavlicek et al. 1999). Treeview (Page 1996) was used to visualize dendrograms. The similarity was calculated according to the Dice coefficient, and dendrograms were constructed based on the unweighted pair-group method with the arithmetic mean (UPGMA). Clustering reliability was checked using Bootstrap and Jackknife methods in FreeTree (Pavlicek et al. 1999). The PIC (polymorphic information content) coefficient was calculated according to the formula (Ghislain et al. 1999) for marker systems of a dominant character: PIC = 1 − *p* ^2^ − *q* ^2^, where *p* is band frequency and *q* is no band frequency. The method efficiency index (EMR) was also determined as the ratio of the number of polymorphic loci to the number of primers/primer combinations used, the differentiation index (DI) as the mean PIC value and the index of the marker system (MI) as the product of the EMR and DI. The analysis of the differences in the distribution of number of the eliminated and new types of bands depending on the line and the triticale generation were performed using the Kruskal–Wallis test (Statistica software ver. 12) with multiple comparisons. Pearson’s linear correlation coefficient was calculated to analyze the correlation between the number of chromosomes and the number of eliminated bands. The test probability was considered significant at *P* < 0.05 and highly significant at *P* < 0.01.

**Table 1:** Sequence of ISSR, IRAP and REMAP primers and their annealing temperatures (Ta)

Methods	Primers	Abbreviations	5′ → 3′ Sequence	Ta
ISSR	(AAC)_6_G IS1	IS1	AACAACAACAACAACAACG	43
	(AC)_10_TA IS2	IS2	ACACACACACACACACACACTA	50
	(ACAG)_5_C	IS3	ACAGACAGACAGACAGACAGC	50
	(CA)_8_GG	IS4	CACACACACACACACAGG	49
	(CAG)_6_A	IS5	CAGCAGCAGCAGCAGCAGA	52
	(CT)_9_A	IS6	CTCTCTCTCTCTCTCTCTA	46.5
	(CT)_9_G	IS7	CTCTCTCTCTCTCTCTCTG	47
	(CTC)_6_G	IS8	CTCCTCCTCCTCCTCCTCG	55
	(CTGT)_5_G	IS9	CTGTCTGTCTGTCTGTCTGTG	52
	(GA)_8_A	IS10	GAGAGAGAGAGAGAGAA	43
	(GA)_8_CT	IS11	GAGAGAGAGAGAGAGACT	46.5
	(GA)_9_C	IS12	GAGAGAGAGAGAGAGAGAC	47
	(GAA)_6_T	IS13	GAAGAAGAAGAAGAAGAAT	42
	(GATA)_2_(GACA)_2_	IS14	GATAGATAGACAGACA	37
	(GGA)_6_T	IS15	GGAGGAGGAGGAGGAGGAT	52
	(GGAA)_5_T	IS16	GGAAGGAAGGAAGGAAGGAAT	52
	(GT)_8_TA	IS17	GTGTGTGTGTGTGTGTTA	42
	(GTA)_6_C	IS18	GTAGTAGTAGTAGTAGTAC	40
	(GTC)_6_A	IS19	GTCGTCGTCGTCGTCGTCA	50
	(GTT)_6_A	IS20	GTTGTTGTTGTTGTTGTTA	42
	(TCT)_6_G	IS21	TCTTCTTCTTCTTCTTCTG	42
	(TGA)_6_A	IS22	TGATGATGATGATGATGAA	40
	(TGC)_6_A	IS23	TGCTGCTGCTGCTGCTGCA	50
IRAP	*Angela*	IR1	GAGGCTCACTAGGGACACAGT	53
	*Sukkula*	IR2	GTCACGCCCAAGATGCGACC	55
	*Bilby*	IR3	GTGCTTGGCGGTTAGCCTCGGCAT	60
	*Cassandra*	IR4	TGCGCACTTTGTCCTCACTCA	52
REMAP		IR1/IS4		50
		IR1/IS9		52
		IR1/IS16		52
		IR1/IS19		50
		IR2/IS3		54
		IR2/IS8		54
		IR2/IS18		46
		IR2/IS19		54
		IR3/IS5		54
		IR3/IS20		46
		IR3/IS21		46
		IR3/IS23		54
		IR4/IS3		50
		IR4/IS4		50
		IR4/IS9		52
		IR4/IS19		50

## Results

### Number of chromosomes in somatic cells of LMUR hybrids

The total number of chromosomes and the proportion of rye and wheat chromosomes was determined in each of the studied lines in two generations. The minimum number recorded was 34, which occurred in LMUR 60F5 (Table 2). In LMUR hybrids, a maximum of 56 chromosomes were observed in some plants from the F5 generation in lines 51, 54, 61 and 107, and in the F8 generation, line 107. The average chromosome number for all lines was 44 chromosomes. The average number of chromosomes in each line was not lower than 40 or higher than 50. The differences in chromosome number distributions only concerned LMUR 60F5 in relation to LMUR 51F5, 107F5 and 107F8. Other differences in chromosome number distribution, including intergenerational differences in one line, were not significant (Table 2). Very little variation was found in the number of rye chromosomes, which always occurred in the number of 10–14 (Table 2). The complete set of rye chromosomes was found from 33% (LMUR 54F8) to 80% (LMUR 54F5) of the tested plants. Such a low variation in the number of chromosomes influenced the absence of significant differences in the distribution of rye chromosomes between individual forms. Chromosome pair 4R and/or 5R were most often missing, most plants with missing chromosomes 4R and/or 5R were found in LMUR 54F8 (40% each). In different lines, there were also plants with no 2R, 3R or 7R chromosome pairs in somatic cells, of which chromosome 3Rs were most often missing.

**Table 2:** The number of chromosomes in somatic cells in five LMUR triticale lines

Triticale lines LMUR	Number of chromosomes						% of cells with			% of cells with eliminated rye chromosomes				
	Total		Rye		Wheat		1RS.1BL chromosome	42 wheat chromosomes	14 rye chromosomes					
	Mean	From-to	Mean	From-to	Mean	From-to				2R	3R	4R	5R	7R
51F5	45.4	38–56	13.5	10–14	32.3	24–42	100	6.9	79.3	–	6.9	17.2	–	–
51F8	43.9	38–50	13.3	12–14	30.6	26–36	70.0	0	65.0	–	15.0	10.0	10.0	–
54F5	46.1	40–56	13.5	10–14	32.7	26–42	53.8	13.3	80.0	–	–	–	20.0	–
54F8	43.9	40–50	12.4	10–14	31.5	26–38	33.3	0	33.3	–	–	40.0	40.0	–
60F5	41.1	34–46	13.2	10–14	27.9	22–32	75.0	0	65.0	5.0	–	15.0	15.0	5.0
60F8	42.0	36–52	13.1	10–14	28.9	24–38	75.0	0	62.5	–	6.3	6.3	31.3	–
61F5	43.9	40–56	13.4	10–14	30.5	26–42	75.0	6.3	75.0	–	–	25.0	6.25	–
61F8	42.3	38–50	12.8	10–14	29.5	26–38	80.0	0	53.3	–	3.3	30.0	23.3	3.3
107F5	46.3	36–56	13.0	12–14	33.3	22–42	61.1	5.6	50.0	–	–	27.8	22.2	–
107F8	46.8	38–56	12.5	10–14	34.0	26–42	66.7	6.7	46.7	–	6.7	13.3	46.7	6.7
Mean LMUR	44.1 ± 4.4	34–56	13.1 ± 1.3	10–14	31.0 ± 4.2	22–42	72.7	4.1	61.9	0.5	4.1	19.1	19.6	1.5

The smallest number of wheat chromosomes was 22 in LMUR 60F5 and LMUR 107F5 plants (Table 2). However, it should be noted that despite the lack of differences in the chromosome number, the chromosome composition might be different because we did not distinguish A, B and D wheat chromosome sets in this study. The highest number was 42 wheat chromosomes (51F5, 54F5, 61F5, 107F5 and 107F8). The average number of wheat chromosomes for all LMUR lines was 31. The differences in the distribution of wheat chromosomes proved to be significant in exactly the same lines as for the total number of chromosomes, indicating that the differences in the number of wheat chromosomes determined the significant differences in the total number of chromosomes. LMUR 60F5 plants had significantly less chromosomes than LMUR 51F5 and highly significantly less compared to both LMUR 107 generations. Unlike rye chromosomes, very few plants contained a complete set of (42) wheat chromosomes in somatic cells. While on average almost 62% of LMUR plants had a complete set of rye chromosomes, a complete set of wheat chromosomes was on average present only in slightly over 4% of plants. The highest number of plants with 42 wheat chromosomes was found in LMUR 54F5, in which the highest number of plants also contained a complete set of rye chromosomes.

Lanca wheat brought to the triticale genome a pair of chromosomes with translocated short arm of wheat chromosome 1R. These chromosomes (1RS.1BL) were not present in all tested LMUR triticale plants (Table 2); it was specific only for LMUR 51F5. In the remaining lines, the percentage of plants with this pair of chromosomes was from 33% in LMUR 54F8 to 80% in LMUR 61F8. The average for all lines was about 73% of plants with translocated wheat chromosomes.

### Analysis of telomeric heterochromatin (t-heterochromatin) quantity in rye chromosomes

**Fig. 1:**
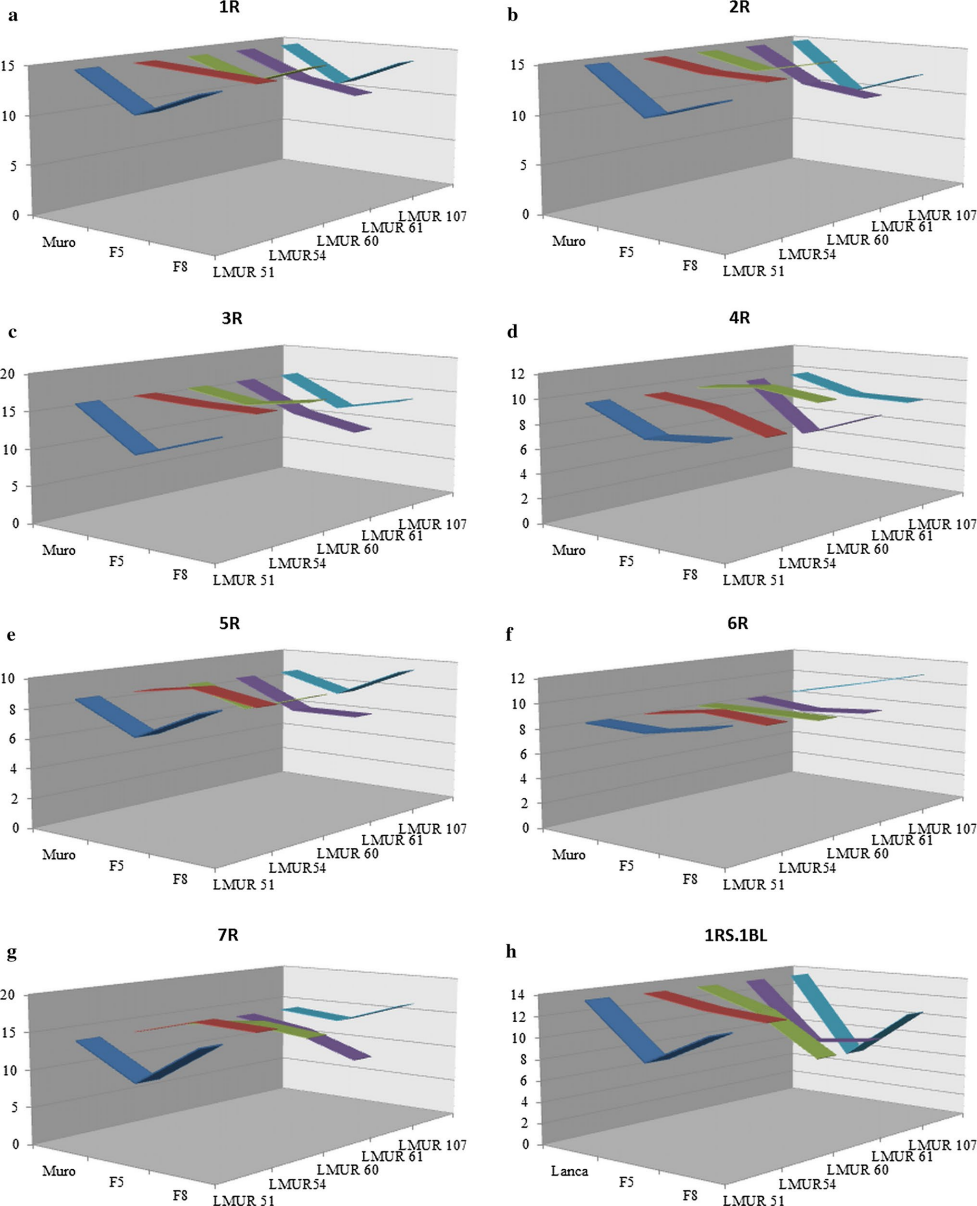
**a**–**h** Changes in t-heterochromatin content (given as a percentage of the total chromosome length) in rye chromosomes 1R (**a**), 2R (**b**), 3R (**c**), 4R (**d**), 5R (**e**), 6R (**f**), 7R (**g**) and in 1RS.1BL chromosome (**h**) of F5 and F8 generations of LMUR triticale lines, in relation to Muro rye (**a**–**g**) and Lanca wheat

**Fig. 2:**
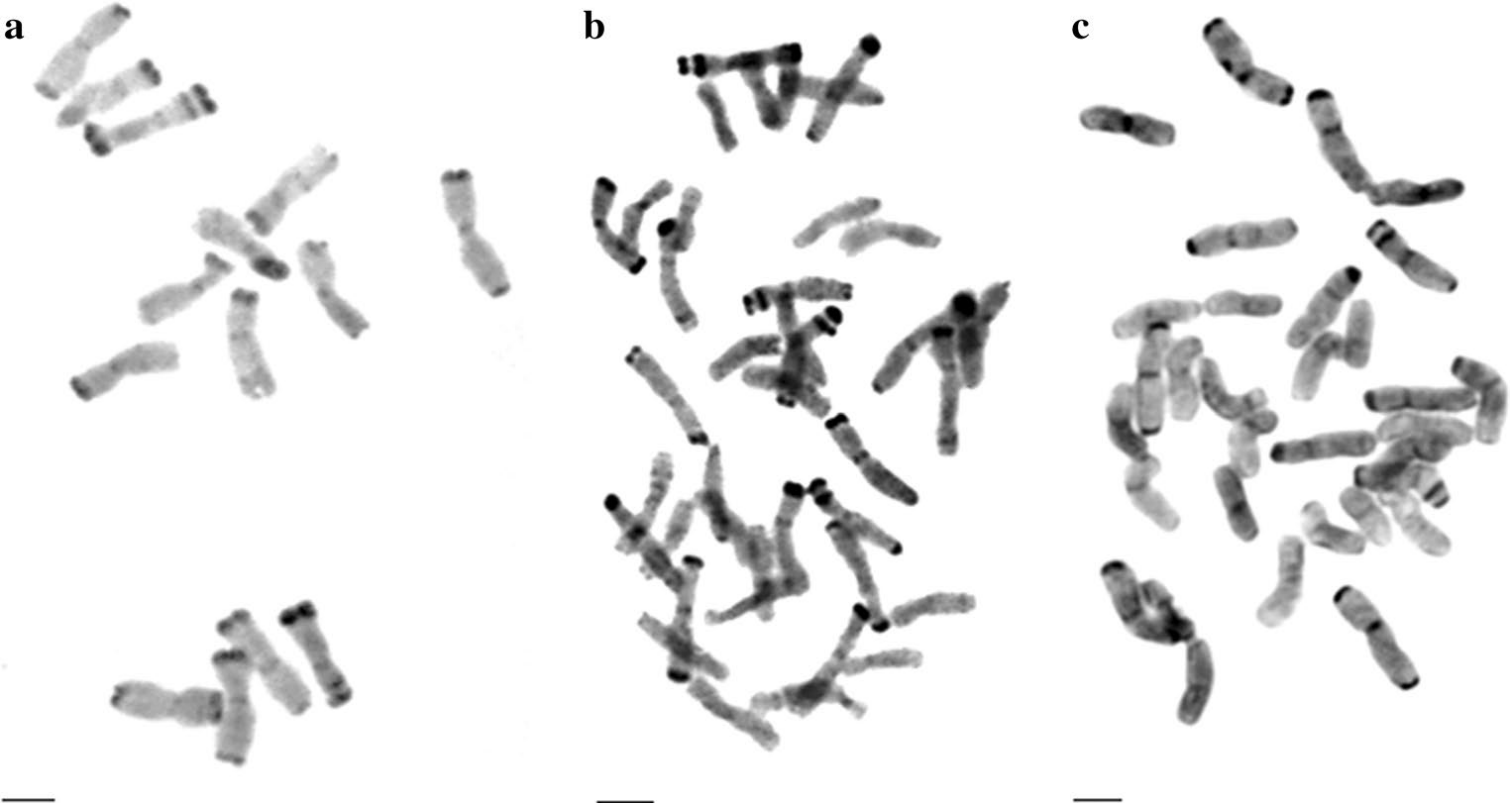
C-banded metaphases of rye (*Secale cereale* L. cv. Muro) (**a**), triticale LMUR 60F5 (**b**) and LMUR 61F5 (**c**). Scale bar 5 µm

There was a decrease in % t-heterochromatin in triticale chromosomes, as compared to parental species (Table 3, Figs. 1, 2). The mean t-heterochromatin content in chromosomes 1R, 2R, and 3R in all triticale lines was lower than in rye, while for all of these chromosomes in LMUR 51F5, 61F8 and 107F5, these differences were significant, similarly for chromosome 2R in LMUR 61F5. In chromosome 4R, t-heterochromatin content was lower in all lines except for LMUR 60F5, and in LMUR 61F5, this difference was significantly lower. In chromosomes 5R, 6R and 7R, the amount of t-heterochromatin in triticale was lower in some lines (LMUR 51F5, 61F8) and higher in several others compared to rye (LMUR 51F8, 54F5, 54F8, 107F8) (Table 3, Fig. 1). Significantly higher t-heterochromatin content compared to rye was found only in chromosome 6R in LMUR 107F8, while significantly lower in chromosome 7R in 51F5 and 61F8. There was less t-heterochromatin in each form of triticale in chromosome 1RS.1BL than in wheat, and was significantly lower in LMUR 51F5, 60F8, 61F5, 61F8 and 107F5 (Table 3, Fig. 1).

**Table 3:** The percentage of t-heterochromatin (th) in chromosomes

Line chromosome		LMUR										
		51F5	51F8	54F5	54F8	60F5	60F8	61F5	61F8	107F5	107F8	Muro
1R	1RS %th	11.36* ± 4.01	18.00 ± 3.63	15.85 ± 3.59	17.31 ± 4.53	10.51 ± 7.80	15.98 ± 2.76	14.94 ± 3.37	11.33* ± 4.04	12.08 ± 3.98	15.79 ± 3.79	15.96 ± 2.76
	1RL %th	10.79 ± 3.49	10.48 ± 4.06	13.12 ± 2.79	11.51 ± 3.37	14.15 ± 3.57	13.64 ± 2.78	10.16 ± 3.98	10.80 ± 3.45	10.02* ± 3.34	12.31 ± 2.52	13.64 ± 2.76
	1R %th	11.03* ± 3.38	13.63 ± 3.09	14.23 ± 1.66	13.81 ± 2.82	12.51 ± 3.94	14.64 ± 2.32	12.09 ± 2.34	11.03* ± 3.38	10.88** ± 3.20	13.73 ± 2.35	14.64 ± 2.32
2R	2RS %th	10.67* ± 1.97	13.09 ± 3.64	14.03 ± 3.56	14.57 ± 3.28	14.35 ± 3.57	15.28 ± 4.52	10.88* ± 2.78	10.67* ± 1.97	8.88** ± 2.73	12.37 ± 3.56	15.40 ± 4.61
	2RL %th	10.77** ± 3.86	12.46 ± 3.09	14.25 ± 3.96	13.53 ± 2.75	13.10 ± 3.97	15.02 ± 3.41	12.05 ± 3.36	10.77** ± 3.86	10.72* ± 4.09	12.01 ± 3.32	14.98 ± 3.50
	2R %th	10.64** ± 2.04	12.69 ± 2.58	14.04 ± 2.64	13.89 ± 2.26	13.59 ± 3.02	14.93 ± 2.62	11.45* ± 2.74	10.64** ± 2.04	9.84** ± 3.02	12.11 ± 2.13	14.96 ± 2.69
3R	3RS %th	9.39** ± 3.52	14.89 ± 3.63	16.16 ± 3.07	14.72 ± 3.51	13.93 ± 5.12	16.74 ± 5.19	12.16 ± 2.52	9.39** ± 3.52	11.15** ± 2.20	13.12 ± 3.43	17.06 ± 3.13
	3RL %th	11.76* ± 3.33	12.66 ± 3.18	15.56 ± 3.80	16.57 ± 3.27	15.49 ± 3.49	15.58 ± 3.53	12.63 ± 3.15	11.76* ± 3.33	12.90 ± 3.74	14.64 ± 3.35	15.64 ± 3.62
	3R %th	10.68** ± 3.07	13.51 ± 1.38	15.71 ± 2.76	15.62 ± 2.49	14.73 ± 3.36	15.96 ± 3.38	12.35 ± 2.63	10.68** ± 3.07	12.00** ± 2.12	13.88 ± 2.61	16.13 ± 3.39
4R	4RS %th	20.11 ± 6.61	21.31 ± 5.51	24.39 ± 7.14	20.58 ± 3.59	27.49 ± 6.53	24.78 ± 5.21	16.87** ± 5.08	20.11 ± 6.61	20.83 ± 5.71	23.44 ± 5.17	25.21 ± 4.99
	4R %th	7.59 ± 2.19	8.02 ± 2.37	9.13 ± 2.55	7.58 ± 1.35	10.26 ± 2.23	9.58 ± 2.11	5.66** ± 1.23	7.59 ± 2.19	8.32 ± 2.27	8.25 ± 2.07	9.74 ± 2.04
5R	5RS %th	17.59 ± 6.53	23.49 ± 5.52	25.04 ± 6.54	24.32 ± 5.47	19.59 ± 6.30	22.09 ± 4.30	19.37 ± 4.61	17.59 ± 6.53	19.24 ± 5.92	25.61 ± 6.92	22.11 ± 4.41
	5R %th	6.73 ± 2.69	8.68 ± 2.68	9.27 ± 2.67	8.50 ± 2.06	7.40 ± 2.13	8.63 ± 1.87	6.71 ± 1.66	6.73 ± 2.69	7.44 ± 2.30	9.40 ± 2.26	8.66 ± 1.92
6R	6RS %th	22.50 ± 6.66	24.66 ± 6.12	24.68 ± 5.45	24.05 ± 5.25	22.48 ± 5.68	23.08 ± 5.91	24.70 ± 7.11	22.50 ± 6.66	24.35 ± 6.21	29.94* ± 4.44	23.21 ± 6.04
	6R %th	8.38 ± 2.43	9.27 ± 2.62	9.24 ± 1.85	8.85 ± 1.66	8.54 ± 2.10	8.50 ± 2.08	8.01 ± 2.27	8.38 ± 2.43	9.65 ± 2.31	10.86* ± 1.56	8.54 ± 2.13
7R	7RS %th	10.27 ± 4.54	21.73 ± 6.77	23.98 ± 6.44	22.90 ± 5.92	16.54 ± 5.25	16.13 ± 5.76	16.25 ± 4.47	10.27 ± 4.54	13.83 ± 5.27	25.68** ± 4.69	16.34 ± 5.84
	7RL %th	9.35 ± 2.51	10.00 ± 2.46	10.45 ± 1.99	10.75 ± 3.04	13.77 ± 4.00	12.48 ± 3.31	9.77 ± 3.45	9.35 ± 2.51	12.76 ± 4.20	8.87 ± 2.22	12.39 ± 3.38
	7R %th	9.69* ± 2.14	15.17 ± 2.73	16.29 ± 3.31	15.91 ± 2.01	14.92 ± 3.76	13.99 ± 3.12	12.67 ± 3.41	9.69* ± 2.14	13.23 ± 3.43	16.23 ± 2.47	14.03 ± 3.21
1RS.1BL	1RS %th	9.42** ± 3.64	18.27 ± 8.36	17.95 ± 3.89	15.92 ± 3.50	13.90 ± 8.41	16.14 ± 3.48	16.26 ± 4.11	9.42** ± 3.64	9.19** ± 7.88	18.76 ± 3.25	18.86 ± 3.53
	1BL %th	8.05 ± 2.58	5.66 ± 4.23	9.03 ± 4.41	8.94 ± 2.68	10.11 ± 4.63	1.35** ± 1.92	1.61** ± 2.69	8.05 ± 2.58	3.42** ± 1.97	4.46* ± 2.96	9.51 ± 3.10
	1RS.1BL %th	8.60** ± 2.45	11.62 ± 2.55	12.49 ± 2.80	11.99 ± 2.15	11.60 ± 4.54	7.85** ± 1.78	7.87** ± 2.57	8.60** ± 2.45	5.76** ± 3.00	10.53 ± 2.17	13.50 ± 2.50

The standard content given in the last column—in Muro rye for chromosomes 1R–7R and in Lanca wheat for 1RS.1BL. Significant/highly significant differences are marked between LMUR hybrid lines and Muro rye/Lanca wheat

* Probability test, *P* < 0.05, ** probability test, *P* < 0.01

Changes in the percentage of t-heterochromatin content also depended on the line, the greatest changes occurred in lines 51 and 107. There were no significant differences between the generations except for line 51 with respect to t-heterochromatin content in chromosome 7R (Table 3, Fig. 1).

Based on the analysis of changes in t–h quantity in all chromosomes of all lines, it could be noted that the amount of t-heterochromatin in the F5 generation was much lower, as compared to the parental species, but it was increasing in the F8 generation and was again more similar to that of the parental species, however, often slightly lower. Such a regularity—less chromosome heterochromatin in triticale in the F5 generation than in the parental species and more in F8 than in F5—was observed in LMUR 51 for all wheat chromosomes and 1RS.1BL, in LMUR 60 for 1R–3R and 5R, in LMUR 61 for 4R–6R and 1RS.1BL, in LMUR 107 for 1R–3R, 5R, 7R and 1RS.1BL. The amplitude of these changes was different, often very high in LMUR 51 and 107, lower in LMUR 60 and 61.

### Occurrence and location of selected retrotransposons

**Fig. 3:**
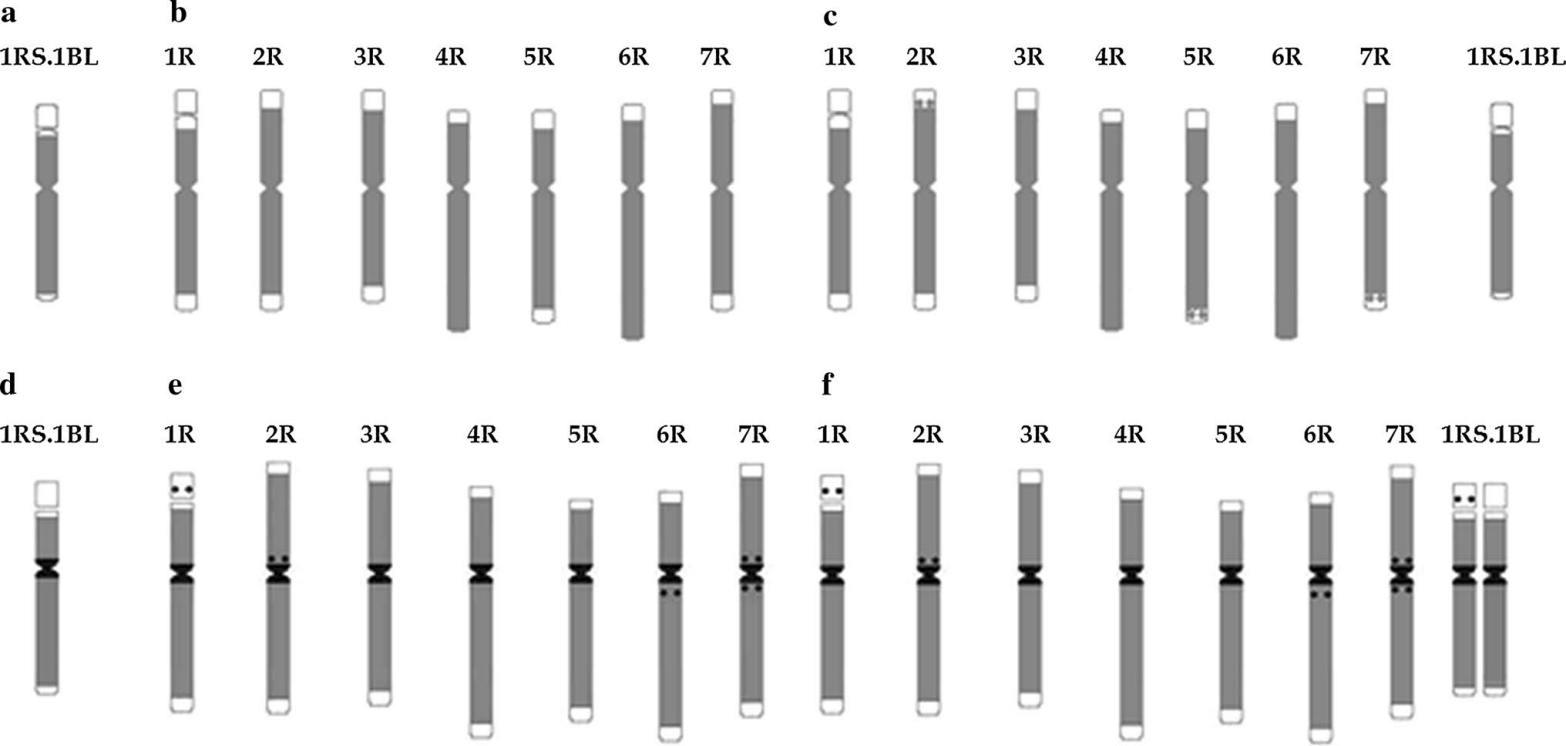
Idiograms showing location of retrotransposons of the family *Ty1*-*copia* (gray color) in common wheat cultivar Lanca on chromosome with translocated rye short arm 1R (**a**), in rye cultivar Muro on all chromosomes in the genome (**b**), in LMUR triticale on chromosomes from the rye genome and wheat chromosome with the translocated short arm of rye chromosome 1R (**c**) and *Ty3*-*gypsy* (gray color, regions with the highest number of copies are indicated in black) in common wheat cultivar Lanca on chromosome 1RS.1BL (**d**), in rye cultivar Muro on all chromosomes in the genome (**e**), in LMUR triticale on chromosomes from the rye genome and wheat chromosome with the translocated short arm of rye chromosome 1R (**f**)

**Fig. 4:**
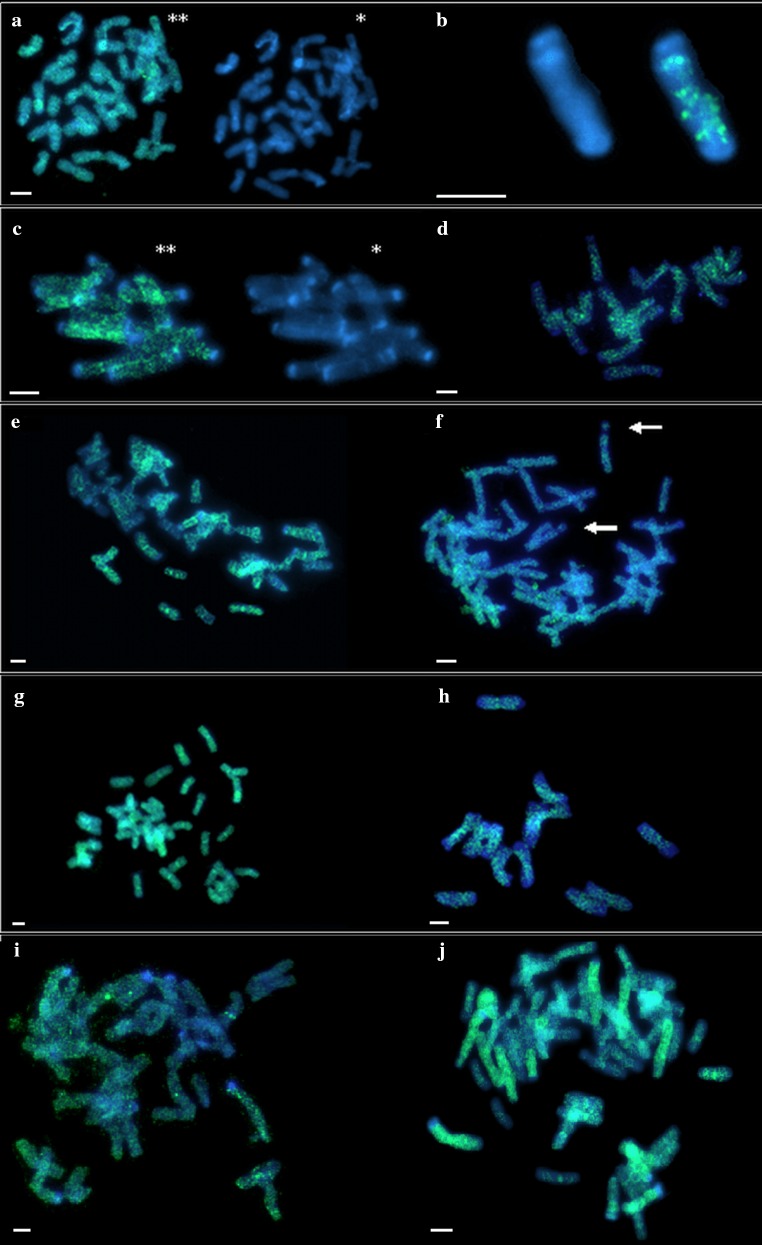
Comparative *Ty1*-*copia* and *Ty3*-*gypsy* in situ hybridization analysis in rye, wheat and triticale after DAPI staining. Asterisk, DAPI image; double asterisk, DAPI image with superimposed location of the probe; somatic cell metaphase chromosomes. **a**–**f** *Ty1*-*copia* in situ hybridization. **a** Lanca wheat metaphase plate. **b** Wheat chromosome with 1RS translocation in common wheat cultivar Lanca. **c**, **d** Metaphase plates of rye cultivar Muro. **e** LMUR triticale metaphase plate, visible local density of retrotransposons in centromeres. **f** LMUR triticale metaphase plate, arrows indicate chromosome 1R satellites containing retrotransposons. **g**–**j** *Ty3*-*gypsy* in situ hybridization. **g** Metaphase plate of common wheat cultivar Lanca. **h** Metaphase plates of rye cultivar Muro. **i**, **j** LMUR triticale metaphase plates. Scale bar 5 µm

The presence of *Ty1*-*copia* and *Ty3*-*gypsy* retrotransposons was determined in F5 and F8 generations of LMUR triticale lines 51, 54, 60, 61 and 107 as well as in Muro rye and Lanca wheat. The presence of retrotransposon families tested was confirmed in all lines on the basis of the expected PCR product (reverse transcriptase gene fragment). With regard to the family *Ty1*-*copia* in the LMUR line, Muro rye and Lanca wheat, the fragment was about 272 bp in size, while the presence of the family *Ty3*-*gypsy* was confirmed by obtaining a fragment of about 422 bp. *Ty1*-*copia* retrotransposons in Muro rye and Lanca wheat were distributed throughout the length of the chromosomes, with regions of lower or higher concentration in all chromosomes, also in centromeres, except for large heterochromatic blocks (Figs. 3, 4). In wheat chromosome 1RS.1BL (Fig. 3a), retrotransposons occurred along the entire length of the chromosome, with the exception of t-heterochromatin, secondary constriction and in its vicinity, and the satellite. In rye chromosome 1R, no retrotransposons were observed in t-heterochromatin, secondary constriction and in its vicinity, except that this area was slightly larger than in the 1RS arm of chromosome 1RS.1BL. *Ty1*-*copia* retrotransposons did not occur in the t-heterochromatin regions of 2R, 3R, and 7R chromosomes. 4RS, 5RS, 6RS did not have retrotransposons in the t-heterochromatin and 5RL in the telomeric and subtelomeric regions (Fig. 3b). In most triticale plants, a similar distribution pattern was observed as in rye in chromosomes 1R–7R and in wheat in 1RS.1BL, but additional hybridization signals were observed in the proximal region in 2RS, 5RL and 7RL in some plants (Figs. 3c, 4). In other plants, the pattern of retrotransposon distribution was identical to that of parental forms. In several plants, there were an increased number of signals in the centromeres of some chromosomes, and signals were even observed in the satellite region in certain plants (Fig. 4f).

The general pattern of retrotransposon distribution of the family *Ty3*-*gypsy* was similar for all chromosomes. They were present along the entire length of the chromosome except for the t-heterochromatin region; however, with significantly higher number of copies within the centromere. In wheat chromosome 1RS.1BL, retrotransposons were found throughout the length of the chromosome, with the most intense hybridization signals occurring in the centromere (Figs. 3d, 4). In all rye chromosomes, retrotransposons were localized similarly throughout chromosomes with local density around centromeres (Figs. 3e, 4). More retrotransposons were also observed near centromeres on short arms 2R and 7R and long 6R and 7R. Clear hybridization signals were found in the satellite of chromosome 1R. No retrotransposons were found in any of the large blocks of t-heterochromatin and even in the small bands in 4RL, 5RL and 6RL. Hybridization signals on rye chromosomes in LMUR triticale were arranged identically as in Muro rye. In triticale chromosome 1RS.1BL, the same pattern of retrotransposon distribution was observed in some plants, as in wheat, and in others there were clear signals in the satellite, similarly to rye chromosome 1R (Figs. 3f, 4).

### Sequence rearrangement analysis: ISSR, IRAP and REMAP

**Fig. 5:**
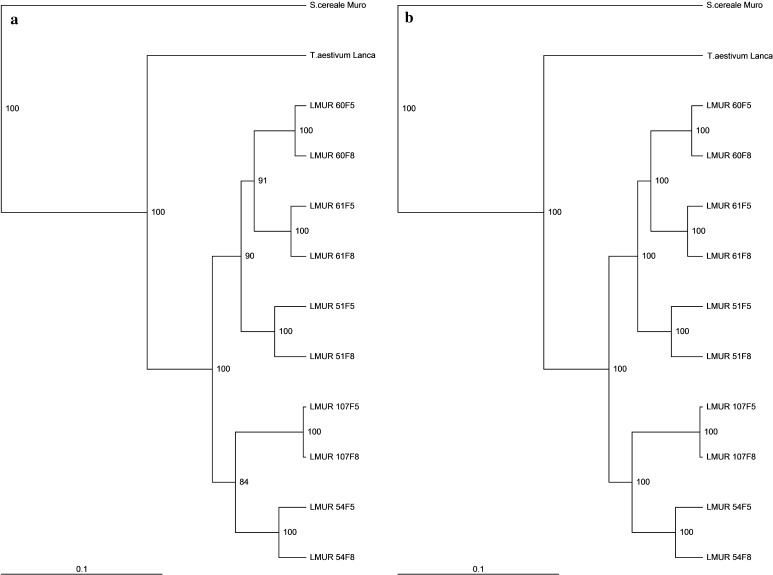
Dendrograms based on band patterns obtained by ISSR, IRAP and REMAP methods, showing similarity of LMUR triticale lines and its parental form, Lanca wheat and Muro rye, including data showing the reliability of clustering. **a** Bootstrap method. **b** Jackknife method

A complex of three methods was used to evaluate the sequence rearrangement in the triticale genome to assess the scale of the reconstruction of the hybrid genome as accurately as possible. By using ISSR primers, 326 DNA fragments were obtained; the individual primers amplified from 11 (IS13) to 28 (IS11) DNA fragments. There were 244 (74.84%) polymorphic products obtained, with an average EMR of 12.2 per primer. The level of polymorphism (PIC) for the individual ISSR primers ranged between 0.13 and 0.27 (Table 4). The 4 different IRAP reactions allowed to obtain 104 bands in total, of which 92 were polymorphic, with an average of 23 per primer (EMR) (Table 5). In total, from 21 (IR1) to 32 (IR3) bands were obtained in reactions. The PIC value for particular reactions was in the range of 0.19–0.26, with the average value of this index (DI) amounting to 0.24 (Table 5). In 16 REMAP reactions, a total of 247 bands was obtained, 185 of which were polymorphic (88.46%) (Table 6). In particular reactions, from 8 (IR1/IS9) to 21 (IR4/IS4) bands were obtained. The PIC value for particular reactions was between 0.13 and 0.25. The IRAP method showed the highest values of DI, EMR and eventually MI, while MI for REMAP was 1.95, for ISSR 2.42, and in IRAP reactions it was 5.46. This is due, in part, to the fact that only four primers giving the best results were selected in the IRAP method, while in ISSR and REMAP methods, there were also other primers, providing slightly worse results. If four primers giving the best results were selected, the results of all methods would be comparable, so these three methods are ideally suited to investigate genomic changes in triticale. The total analysis of all ISSR, IRAP and REMAP reaction results showed 44% similarity between the band patterns of wheat and rye (Table 7). Line-dependent band patterns of LMUR triticale were 77–84% similar to wheat lines. Line 54 and line 107 showed the highest similarity. The lowest similarity was observed in line 60. Triticale band patterns showed 60–67% similarity in relation to rye. Line 51 and line 61 showed the highest similarity. The similarity between the generations of a single line ranged from 96% in LMUR 51–100% in LMUR 107. Lines LMUR 54 and 107 clustered together (Fig. 5), while the second cluster was formed by LMUR lines 51, 60 and 61. Bootstrap and Jackknife analyses (Fig. 5) showed a high clustering reliability. There were 8 different trees generated in the Bootstrap analysis, of which the reference tree appeared 763 times; in the Jackknife analysis, only reference trees were present.

**Table 4:** Characteristics of the ISSR markers polymorphism

No.	Primer	Number of fragments		% Polymorphic fragments	PIC	Product size range (bp)
		Total	Polymorphic			
1	IS1	12	7	58.33	0.14	547–1159
2	IS2	13	9	69.23	0.13	285–971
3	IS3	25	19	76.00	0.19	326–1860
4	IS4	20	16	80.00	0.19	550–1665
5	IS5	14	8	57.14	0.21	201–666
6	IS6	13	10	76.92	0.27	398–1398
7	IS7	18	13	72.22	0.16	445–2506
8	IS9	15	12	80.00	0.21	408–1655
9	IS10	13	9	69.23	0.22	414–1434
10	IS11	28	24	85.71	0.27	394–2181
11	IS12	15	12	80.00	0.19	442–1189
12	IS13	11	8	72.73	0.14	278–831
13	IS14	21	16	76.19	0.21	263–2161
14	IS15	16	8	50.00	0.15	238–893
15	IS16	14	11	78.57	0.25	318–1108
16	IS17	14	12	85.71	0.20	475–1501
17	IS19	16	12	75.00	0.18	239–872
18	IS20	20	15	75.00	0.23	278–1522
19	IS21	15	13	86.67	0.18	379–1419
20	IS22	13	10	76.92	0.21	350–901
	Mean	16.3	12.2	74.10	0.20	–
	Min–max	11–28	7–24	50.00–86.67	0.13–0.27	201–2506

**Table 5:** Characteristics of the IRAP markers polymorphism

No.	Primer	Number of fragments		% Polymorphic fragments	PIC	Product size range (bp)
		Total	Polymorphic			
1	IR1	21	19	90.48	0.23	272–1375
2	IR2	26	22	84.61	0.19	147–1335
3	IR3	32	28	87.50	0.26	203–3195
4	IR4	25	23	92.00	0.26	280–1995
	Mean	26	23	88.65	0.24	–
	Min–max	21–32	19–28	87.50–92.00	0.19–0.26	147–3195

**Table 6:** Characteristics of the REMAP markers polymorphism

No.	Primer	Number of fragments		% Polymorphic fragments	PIC	Product size range (bp)
		Total	Polymorphic			
1	IR1/IS4	18	12	66.67	0.16	287–1254
2	IR1/IS9	8	6	75.00	0.17	132–798
3	IR1/IS16	17	10	58.82	0.13	136–1210
4	IR1/IS19	18	14	77.78	0.13	261–1172
5	IR2/IS3	14	8	57.14	0.13	170–839
6	IR2/IS8	15	8	53.33	0.14	132–1213
7	IR2/IS18	10	8	80.00	0.15	274–656
8	IR2/IS19	18	12	66.67	0.17	230–1219
9	IR3/IS5	16	11	68.75	0.14	206–688
10	IR3/IS20	10	8	80.00	0.17	91–655
11	IR3/IS21	12	9	75.00	0.14	245–1042
12	IR3/IS23	15	12	80.00	0.19	205–1070
13	IR4/IS3	19	15	78.95	0.14	226–1281
14	IR4/IS4	21	19	90.48	0.23	247–1313
15	IR4/IS9	19	18	94.74	0.25	211–1071
16	IR4/IS19	17	15	88.24	0.24	326–974
	Mean	15.44	11.56	74.47	0.17	–
	Min–max	8–21	6–19	53.33–94.74	0.13–0.25	91–1313

**Table 7:** Dice coefficient similarity matrix based on the ISSR, IRAP and REMAP markers polymorphism

	Lanca	Muro	LMUR 51F5	LMUR 51F8	LMUR 54F5	LMUR 54F8	LMUR 60F5	LMUR 60F8	LMUR 61F5	LMUR 61F8	LMUR 107F5	LMUR 107F8
Lanca		0.44	0.79	0.78	0.83	0.84	0.78	0.77	0.80	0.78	0.83	0.83
Muro	0.44		0.67	0.65	0.64	0.61	0.66	0.65	0.66	0.65	0.60	0.60
LMUR 51F5	0.79	0.67		0.96	0.92	0.91	0.93	0.92	0.94	0.93	0.87	0.87
LMUR 51F8	0.78	0.65	0.96		0.91	0.90	0.91	0.90	0.91	0.91	0.86	0.86
LMUR 54F5	0.83	0.64	0.92	0.91		0.97	0.90	0.89	0.91	0.90	0.91	0.90
LMUR 54F8	0.84	0.61	0.91	0.90	0.97		0.88	0.87	0.90	0.88	0.92	0.92
LMUR 60F5	0.78	0.66	0.93	0.91	0.90	0.88		0.99	0.94	0.94	0.87	0.87
LMUR 60F8	0.77	0.65	0.92	0.90	0.89	0.87	0.99		0.93	0.93	0.86	0.86
LMUR 61F5	0.80	0.66	0.94	0.91	0.91	0.90	0.94	0.93		0.98	0.89	0.89
LMUR 61F8	0.78	0.65	0.93	0.91	0.90	0.88	0.94	0.93	0.98		0.87	0.87
LMUR 107F5	0.83	0.60	0.87	0.86	0.91	0.92	0.87	0.86	0.89	0.87		1.00
LMUR 107F8	0.83	0.60	0.87	0.86	0.90	0.92	0.87	0.86	0.89	0.87	1.00

**Fig. 6:**
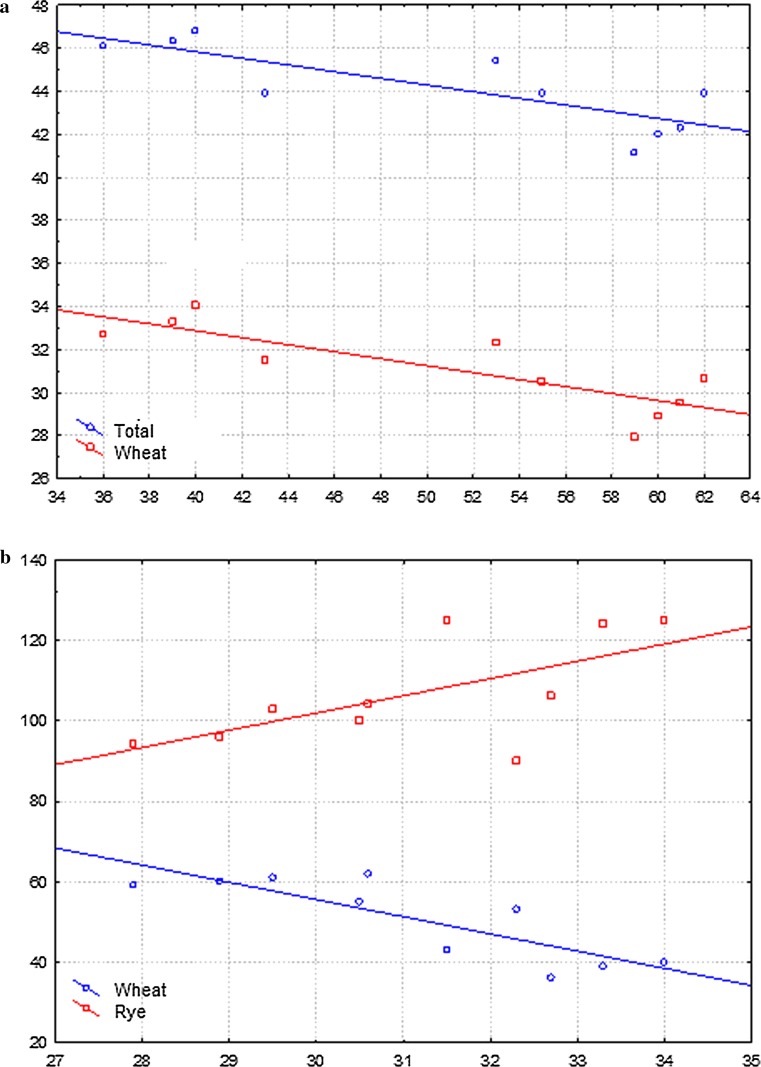
**a** Correlation between the total chromosome number and the number of wheat chromosomes and the eliminated wheat bands (*x* axis: the number of eliminated wheat bands; *y* axis: the number of chromosomes). **b** Correlation between the number of wheat chromosomes and the eliminated wheat and rye bands (*x* axis: wheat chromosome number; *Y* axis: number of eliminated bands)

In total, 677 bands were observed in all ISSR, IRAP and REMAP reactions, of which 248 were present in Lanca wheat (and alternatively triticale), 220 in Muro rye (and alternatively triticale), 186 both in wheat and rye (and alternatively triticale), and 23 types of bands were found exclusively in triticale; they were absent in parental species. On average, 16.9 types of bands were found per reaction, 36.6% of which were bands characteristic for wheat, 32.5% for rye, 27.5% common for wheat and rye and 3.4% found exclusively in triticale (Table 8). The appearance of new types of bands in triticale, absent in parental species, was significantly less frequent than band elimination. Out of a total number of 677 band types, the elimination of 268 bands was observed, which involved 32.5% of wheat bands, 56.7% of rye and 10.8% common for wheat and rye (Table 8). There were more eliminated rye bands than wheat bands in all analyzed lines. Least wheat bands (36–43) were eliminated in LMUR 54 and 107, more (about 53–62) in LMUR 51, 60 and 61 (Table 9). The largest number of eliminated rye bands (106–125) was found in lines 107 and 54. The lowest degree of elimination concerned the bands common for wheat and rye. The largest number of eliminated common bands was found in LMUR 60 (16–18) (Table 9). The highest number of (10) new types of bands (not found in wheat and rye) in triticale was observed in LMUR 60F8 (Table 9). There were no significant differences between the lines of triticale in the number of eliminated bands as well as new wheat, rye and common bands. The lack of significant differences between LMUR triticale lines indicated that bands common to wheat and rye were eliminated in the lowest percentage. This result likely indicates that rye genomic sequences were more prone to rearrangement or possibly elimination. Correlation analysis was performed to verify whether the number of eliminated types of bands was related to the number of chromosomes in individual lines (Table 10; Fig. 6). The number of eliminated wheat bands was negatively correlated with the total number of chromosomes and the number of wheat chromosomes, which meant that the smaller the number of wheat chromosomes in a given line, the higher the number of eliminated wheat bands. Likewise, the lower the number of rye chromosomes in a given line, the higher the number of eliminated rye bands. Positive correlation was found between the number of eliminated rye bands and the number of wheat chromosomes, which indicated that the higher the number of wheat chromosomes in a given line of triticale, the greater the elimination of rye bands.

**Table 8:** Summary of band types and types of eliminated bands in ISSR, IRAP and REMAP analyses

Primer	The number of types of bands									The number of types of eliminated bands						
	Total	Lanca		Muro		Common		New		Total	Lanca		Muro		Common	
		L	%	L	%	L	%	L	%		L	%	L	%	L	%
ISSR	326	126	38.65	94	28.83	97	30	9	2.8	128	51	40	63	49.2	14	10.93
IRAP	104	35	33.65	44	42.3	19	18.27	6	5.77	56	13	23.21	36	64.29	7	12.5
REMAP	247	87	35.22	82	33.2	70	28.34	8	3.24	84	23	27.38	53	63.1	8	9.52
Total	677	248	–	220	–	186	–	23	–	268	87	–	152	–	29	–
Mean	16.9	6.2	36.6	5.5	32.5	4.6	27.5	0.6	3.4	6.7	2.2	32.5	3.8	56.7	0.7	10.8

L indicates the number of band types. In addition, the total number of types/eliminated types of bands for all primers/primer combinations and mean values for single reactions are given

**Table 9:** Summary of eliminated bands and new types of bands in individual LMUR triticale lines determined on the basis of electrophoresis images obtained by the electrophoresis of PCR products in ISSR, IRAP and REMAP analyses

Line	Generation	ISSR		IRAP		REMAP		Cumulatively	
		Total	Mean	Total	Mean	Total	Mean	Total	Mean
LMUR51F5	− L	27	1.35	9	2.25	17	1.06	53	1.3
	− M	38	1.9	22	5.5	30	1.875	90	2.3
	− W	4	0.2	2	0.5	2	0.125	8	0.2
	+ N	3	0.15	2	0.5	0	0	5	0.1
LMUR51F8	− L	35	1.75	10	2.5	17	1.06	62	1.6
	− M	42	2.1	27	6.75	35	2.19	104	2.6
	− W	6	0.3	2	0.5	3	0.19	11	0.3
	+ N	2	0.1	1	0.25	3	0.19	6	0.2
LMUR54F5	− L	22	1.1	4	1	10	0.625	36	0.9
	− M	44	2.2	25	6.25	37	2.31	106	2.7
	− W	3	0.15	2	0.5	1	0.06	6	0.2
	+ N	2	0.1	1	0 25	0	0	3	0.1
LMUR54F8	− L	24	1.2	6	1.5	13	0.81	43	1.1
	− M	52	2.6	30	7.5	43	2.69	125	3.1
	− W	5	0.25	3	0.75	1	0.06	9	0.2
	+ N	4	0.2	1	0.25	0	0	5	0.1
LMUR60F5	− L	30	1.5	10	2.5	19	1.19	59	1.5
	− M	41	2.05	20	5	33	2.06	94	2.4
	− W	8	0.4	4	1	4	0.25	16	0.4
	+ N	2	0.1	2	0.5	0	0	4	0.1
LMUR60F8	− L	31	1.55	10	2.5	19	1.19	60	1.5
	− M	41	2.05	22	5.5	33	2.06	96	2.4
	− W	8	0.4	5	1.25	5	0.31	18	0.5
	+ N	3	0.15	3	0.75	4	0.25	10	0.3
LMUR61F5	− L	31	1.55	10	2.5	14	0.875	55	1.4
	− M	45	2.25	23	5.75	32	2	100	2.5
	− W	4	0.2	2	0.5	2	0.125	8	0.2
	+ N	2	0.1	3	0.75	1	0.06	6	0.2
LMUR61F8	− L	35	1.75	11	2.75	15	0.94	16	1.5
	− M	45	2.25	24	6	34	2.125	103	2.6
	− W	7	0.35	3	0.75	3	0.187	13	0.3
	+ N	2	0.1	4	1	1	0.06	7	0.2
LMUR107F5	− L	23	1.15	6	1.5	10	0.625	39	1.0
	− M	55	2.75	26	6.5	43	2.7	124	3.1
	− W	5	0.25	5	1.25	3	0.19	13	0.3
	+ N	6	0.3	2	0.5	0	0	8	0.2
LMUR107F8	− L	23	1.15	6	1.5	11	0.69	40	1.0
	− M	56	2.8	26	6.5	43	2.69	125	3.1
	− W	5	0.25	5	1.25	3	0.187	13	0.3
	+ N	6	0.3	2	0.25	0	0	8	0.2

L indicates the number of band types eliminated from the wheat genome (Lanca). − M indicates the number of band types eliminated from the rye genome (Muro). − W indicates the number of eliminated band types from the wheat and rye genome. + N indicates the number of new types of bands absent in parental genomes of triticale

**Table 10:** Correlation (Pearson’s correlation coefficient) between the number of chromosomes and the number of eliminated wheat bands (− L), rye bands (− M) and bands common for wheat and rye (− W)

The number of bands	The number of chromosomes		
	Total	Rye	Wheat
− L	− 0.8085**	0.2574	− 0.8324**
− M	0.5539	− 0.7456*	0.6416*
− W	− 0.5474	− 0.2995	− 0.5146

* Probability test, *P* < 0.05, ** probability test *P* < 0.01

The analysis of the rearrangement between F5 and F8 generations in the studied triticale lines (Table 11) showed the least changes between LMUR 107 generations. In this case, there were 176 band types in the F5 generation, and 178 in the F8 generation. There were 8 new band types in F5, and this number did not increase in F8. Thus, the two generations differed in total by only two types of bands. Eleven differentiating bands were observed in LMUR 60, and 15 in LMUR 61. The highest number of differences was found in LMUR 51 and 54, 33 and 30, respectively. Line 51 had a special situation, as the two new types observed in F5 were not present in F8, which was classified as a difference (Table 11).

**Table 11:** Summary of eliminated and new types of bands in individual LMUR triticale lines in two generations, determined on the basis of electrophoresis images resulting from the separation of products of PCR reactions from ISSR, IRAP and REMAP analyses

Line	Generation	ISSR		IRAP		REMAP		Total			
								Total	Number of different bands		
		Total	Number of different bands	Total	Number of different bands	Total	Number of different bands		Between generations	In line	Mean
LMUR 51	F5 −	70	16	33	6	49	6	152	28	33	18.2
	F8 −	86		39		55		180			
	F5 +	3	1	2	1	0	3	5	5		
	F5 +	2		1		3		6			
LMUR 54	F5 −	69	11	31	8	48	9	148	28	30	
	F8 −	80		39		57		176			
	F5 +	2	2	1	0	0	0	3	2		
	F5 +	4		1		0		5			
LMUR 60	F5 −	80	1	34	3	56	1	170	5	11	
	F8 −	81		37		57		175			
	F5 +	2	1	2	1	0	4	4	6		
	F5 +	3		3		4		10			
LMUR 61	F5 −	81	7	35	3	48	4	164	14	15	
	F8 −	88		38		52		178			
	F5 +	2	0	3	1	1	0	6	1		
	F5 +	2		4		1		7			
LMUR 107	F5 −	83	1	37	0	56	1	176	2	2	
	F8 −	84		37		57		178			
	F5 +	6	0	2	0	0	0	8	0		
	F5 +	6		2		0		8			

F5 − indicates the number of eliminated types of bands that were found in the F5 generation. F8 − indicates the number of eliminated band types that were found in the F8 generation. F5 + indicates the number of new types of bands found in the F5 generation. F8 + indicates the number of new types of bands found in the F8 generation

## Discussion

Transformations of parental genomes in newly formed allopolyploids are a multidimensional phenomena, difficult to capture both in time and space. These changes can be slightly different in various species, thus one can only discuss certain tendencies using more or less specific temporal patterns. It is difficult to identify all levels of genomic changes, and very erroneous conclusions can be drawn from partial analyses. The more complete the multidimensional picture, obtained by means as different as possible, allowing to judge from different angles, the more true it is.

In this study, changes in the number of chromosomes and consequently the elimination of many nucleotide sequences were observed in the studied line of wheat–rye hybrids. Changes in the number of chromosomes are mainly due to the meiotic instability (Kalinka et al. 2010). Meiotic disorders lead to changes in chromosome number, which are associated with chromosome segregation disorders, chromatin degradation or chromosome fragmentation in subsequent plant generations. In octoploid triticale, which is cytologically highly unstable (Lukaszewski and Gustafson 1987), the percentage of aneuploid plants is high. The average percentage of aneuploids in the primary triticale lines was high and amounted to 83%, with fluctuations between 30 and 100% (Krolow 1962). Both rye and wheat chromosomes (Merker 1971) contributed to aneuploidy, as opposed to the results reported by Müntzing (1957) and Stutz (1962), who concluded that meiotic disorders and aneuploidy of triticale mainly involved rye chromosomes. Weimarck (1974) pointed out that the proportion of eliminated rye and wheat chromosomes was about 1:3. Since the chromosome ratio in the rye and wheat genome is also 1:3, it indicates that chromosomes are eliminated in equal proportions. In this work we have found higher elimination of wheat chromosomes than that of rye, taking into account the genomic proportions, since the ratio was on average 1:11. Up to 62% of the plants had a complete set of rye chromosomes, and only about 4% a complete set of wheat chromosomes. There was a tendency in rye chromosomes to eliminate chromosome pairs 4R and 5R, which confirmed that chromosome elimination was probably not accidental (Gustafson 1976) and that there was a trend to eliminate certain chromosomes. Direct chromatin elimination was observed in LMUR allopolyploids from the pollen mother cells at different stages of meiotic process (Kalinka et al. 2010). GISH analysis showed, that both wheat and rye chromatin can be eliminated in this manner.

Reorganization and sequence elimination are common in many newly formed allopolyploid species (Ozkan et al. 2001). In this work, sequence analysis was performed using ISSR, IRAP and REMAP methods. All methods have shown that reorganization of parental genomes occurs in hybrids. The disappearance of certain types of bands was mainly observed in relation to the cumulative band pattern in parental forms. This phenomenon was at least partially related to the loss of certain chromosomes, since it was demonstrated in both wheat bands and wheat chromosomes as well as rye bands and rye chromosomes that such correlation occurred. If, however, all the tested lines lacked certain types of wheat or rye bands, then there must have been other mechanisms, certainly directional and recurrent, because not the same chromosomes were eliminated in each plant. The best evidence for this was that more rye bands were missing, although most of the plants had a complete number of rye chromosomes. In turn, lower number of wheat bands disappeared and at the same time, wheat chromosomes were more frequently eliminated, and only a small proportion of the plants had an entire set of them. It is difficult to say whether this is a specific type of sequence elimination associated with retrotransposition or high genetic changes that largely affected regions of microsatellite sequences and retrotransposons. The most likely cause of the observed changes was the activity of genetic elements or recombination between these sequences (Bento et al. 2008). Such hypothesis is supported by the fact that retrotransposon sequence disappearance has been observed in triticale (Han et al. 2003). Additionally, it has been shown that chromosome condensation disorders (as observed in LMUR triticale) often activate mechanisms, such as DNA recombination and/or DNA damage repair, leading to sequence deletion/modification, as demonstrated in *Drosophila* lines with mutations in genes encoding enzymes involved in chromatin remodeling (Peng and Karpen 2007). It may be that several different mechanisms are responsible for the observed genomic differentiation.

The elimination of retrotransposons may be particularly intense (Han et al. 2003), which in comparison to other sequences appears particularly susceptible to the removal from the genome. In some species, reorganization of the sequences was not a process time-stretched over many generations, it was rapid, and most of the changes occurred directly after F1 hybridization (Liu et al. 1998); in other species, changes were observed only in generation 40 (Buggs et al. 2009). In some allopolyploids, the changes were very severe, as in *Triticum* or *Brassica* (Liu et al. 1998; Ozkan et al. 2001), while, e.g., in *Gossypium* or *Spartina*, rearrangement changes were virtually absent (Liu et al. 2001). In this case, it may be suggested that some species have a high tolerance for genome doubling and interspecific hybridization.

It is believed that the homeologous chromosome differentiation is the primary purpose of these changes. Homeologous genomes are most often subjected to significant differentiation, often through the acquisition/loss of genes and repetitive sequences or chromosomal aberrations (Pikaard 2001). Recombination in all possible lines is supposed to be the main factor responsible for these changes. The consequence of such recombination events is the small and larger genetic changes that are detected in newly formed allopolyploids.

The present work determined the occurrence and location of retrotransposons from *Ty1*-*copia* and *Ty3*-*gypsy* families. The FISH method has demonstrated changes in retrotransposon location in triticale compared to parental species. In general, the distribution of *Ty1*-*copia* and *Ty3*-*gypsy* retrotransposon families in wheat and rye chromosomes was consistent with distribution pattern of these sequences in cereals (Schulman et al. 2004). *Ty1*-*copia* retrotransposons in cereals are distributed relatively evenly across all chromosomes of the genome with locally smaller or greater density, except for the centromere, NOR and telomeres. In the case of *Ty3*-*gypsy*, they are scattered along all chromosomes, they are not present in the NOR and telomere regions, but they show a greater concentration in the centromeric region. Certain changes in retrotransposon distribution were observed in triticale compared to parental species. The presence of the *Ty1*-*copia* retrotransposon family in some blocks of telomeric heterochromatin of rye chromosomes in triticale seemed to be most important. *Ty1*-*copia* retrotransposons were not observed at this location in rye, thus changes in rye chromosomes had to occur.

The rearrangement of sequences as well as changes in retrotransposons locations observed in this work may be reflected in alterations in heterochromatin content in chromosomes. The analysis of the content of t-heterochromatin in rye chromosomes and chromosome 1RS.1BL showed changes in its quantity, mainly a decreasing trend compared to parental chromosomes. The reduction of rye heterochromatin in triticale has already been observed (Merker 1975, 1976; Gustafson and Bennett 1976; Roupakias and Kaltsikes 1977; Rogalska 2005) and mainly concerned chromosome pairs 1R, 3R, 5R and 6R. In this study, the highest number of significant differences in the size of C-bands was found on chromosomes 1R, 2R and 3R. The sizes of heterochromatin bands were compared in two forms (hexaploid and octoploid) of primary triticales and their parental species by Badaeva et al. (1986) and Bolsheva et al. (1986). Badaeva et al. (1986) showed, that specially 1R, 2R and 3R rye chromosomes differ the most between triticale and parental rye varieties. It was found that the octoploid triticale had slightly less t-heterochromatin in parallel with more centromeric heterochromatin than rye. Unfortunately, the authors did not mention which generation of triticale they had analyzed. In our study, we have shown that the heterochromatin content changes is a dynamic process and may change in successive generations of triticale plants. Interestingly, Bolsheva et al. (1986) also showed that the telomeric and intercalary C-bands either increased or decreased in size in wheat chromosomes of triticale in comparison to parental wheat varieties, but the sizes of centromeric bands only increased. Moreover, based on the analysis of both triticale forms, it was found that the C-banding pattern of B-genome chromosomes in triticale changes in a regular manner. This is in consistency with this work, as we have shown there is an overall regularity, that the t-heterochromatin content in rye chromosomes in triticale may decrease in F5 generation, while in F8 generation it is similar to parental rye variety.

Changes in chromosome structure, including the emergence (Burns and Gerstel 1967) and disappearance (May and Appels 1980) of heterochromatin blocks or repetitive DNA rearrangements (Kamm et al. 1995) have been also observed in *Arabidopsis*. Transposition and recombination between retrotransposon sequences can also have a significant effect on the phenotype (Chantret et al. 2005) by influencing the rearrangement of the allopolyploid genome structure (Parisod et al. 2010). In addition, because they affect the expression and function of genes (Lockton and Gaut 2009), changes in their localization may lead to hereditary modifications in the expression of different genes (Slotkin and Martienssen 2007). Moreover, the stress associated with distant crossing and polyploidization leads to epigenetic changes, which allows for both gene silencing and activation through allopolyploidization. The abrogation of silencing state due to allopolyploidization may suggest a massive transposition of genetic mobile elements, although there is no significant evidence for this. In most cases, only structural changes in the genome concerning these sequences are found immediately after hybridization or polyploidization, the most common of which is deletion. Such changes may be caused by various types of recombinations (Grover and Wendel 2010). The presence of “damaged”, incomplete retrotransposon sequences, especially LTR sequences alone, is the evidence that deletions and rearrangements often affect regions where retrotransposons are located (Devos et al. 2002). This is confirmed by the fact that the decreased methylation level in response to stress was accompanied by an increased level of recombination (Boyko et al. 2007). As regards chromosome 1RS.1BL, the presence of *Ty3*-*gypsy* retrotransposons was observed in the satellite in triticale, and they were not present in this location in wheat, as opposed to 1RS in rye. Therefore, the recombination between 1R and 1RS.1BL in triticale is most likely the reason of hybridization signals at this site.

The above-described phenomena associated with the activation of mobile elements or recombination between these sequences may also be reflected in sequence rearrangements analyzed by the marker systems in this work. The use of these techniques allows us to assess changes in the genomic scale and to determine which of the parental genomes are rearranged to a higher extent. This study used three genetic marker systems: IRAP, REMAP (Kalendar et al. 1999) and ISSR which showed genetic changes in the wheat and rye genomes in LMUR hybrids. Sequence rearrangements were also investigated at the global level of the triticale genome using AFLP (amplified fragment length polymorphism) and restriction fragment length polymorphism (RFLP) markers (Ma and Gustafson 2006). Hexaploid and octoploid triticales were analyzed, including parental species, F1 wheat–rye hybrids and several early triticale generations. The results showed that the degree of sequence variation is different in hexaploid and octoploid triticale, while similar in lines with the same ploidy. There were less changes in octoploid than in hexaploid triticale (Ma and Gustafson 2006). On average, 30% band loss was observed in octoploid triticale, while 40% band loss in hexaploid triticale. In this work, the use of the ISSR, IRAP and REMAP method complex showed that about 40% of bands observed in parental species were affected by changes in octoploid triticale. Perhaps the LMUR line is characterized by a slightly lower stability than octoploid lines used in other studies. Heterogeneity dependent on the cross-combination of the newly synthesized wheat–rye lines is highly probable. On the other hand, the methods used in this work mainly allowed for the analysis of changes in regions rich in microsatellite sequences and/or rich in retrotransposons, hence the degree of sequence rearrangement could be higher. This would be consistent with the commonly observed model of sequence evolution in the genome, where repetitive sequences are primarily affected by changes and coding sequences show relatively higher conservation, which can be demonstrated by investigating close or more distant related taxa.

It was observed in this study that most of the changes, in the approach using marker systems, involved loss of bands present in the parental species of triticale. Significantly less frequent were the incidences of new types of bands not observed in any of the parental species. Most of the changes in triticale in the above-mentioned studies also more frequently involved loss of bands than the emergence of new ones, similarly as in wheat allopolyploids (Feldman and Levy 2005).

The analyses carried out in this work also indicated that more changes occurred in the rye than wheat genome in triticale, which confirmed the results obtained in other studies (Ma and Gustafson 2006). It can be assumed that the genome of the paternal form is more prone to changes, because it is located in a foreign environment of maternal cytoplasm. The situation, in which one parental genome is adapting better does not concern all allopolyploid species (Liu et al. 1998; Ozkan et al. 2001). There may be several reasons for this variation, such as the degree of relationship between the combined genomes or even the level of their ploidy. Another possible explanation for the fact that the rye genome undergoes greater transformations may be that hexaploid wheat has already experienced allopolyploidization in its evolutionary history as opposed to diploid rye, and thus its genome is more suited to functioning in a hybrid.

Bento et al. (2008) conducted experiments using ISSR, IRAP and REMAP methods in octoploid triticale and showed the elimination of approximately 43% of the observed bands. New bands present in triticale and absent in any parental species accounted for 7%. The results obtained in this study were very similar, because the total number of eliminated bands out of all observed bands was 40%, while new bands represented slightly over 3% of all observed bands. Both studies found the elimination of a greater percentage of rye bands compared to wheat bands. However, in contrast to Bento et al. (2008), the loss of bands common for wheat and rye was observed in our study. Both studies found a very high percentage of polymorphic bands, which in the study of Bento et al. (2008) was 65% for IRAP and REMAP methods and 68% for ISSR, while in the current study it was 74% for ISSR and REMAP and 89% for IRAP. This confirms that the combination of these three marker systems is an excellent tool for testing closely related genomes.

These analyses demonstrated that most of the genetic changes occurred in the F5 generation, with fewer changes in the period between the F5 and F8 generation. Literature indicates that most of the changes observed in triticale appear already in F1 hybrids, even before chromosome doubling (Ma and Gustafson 2006). Changes in successive generations after chromosome doubling in triticale were relatively lower and their level equalized from generation to generation (Ma and Gustafson 2006). However, as shown by the results of this work, it may be line-dependent and there may be relatively many changes also in later generations. In the study conducted by Ma and Gustafson (2006), changes in the number of eliminated wheat bands were difficult to detect, as such elimination was relatively rare. The loss of rye bands was noticeable in the early generations of triticale, and the proportion of the lost rye bands increased to the F5 generation. While F1 hybrids showed 68.7% elimination of wheat bands, in F1 triticale it was 0.2, 1.1% in F2, 4.0% in F3, 4.0% in F4 and 8.1% in F5. The remaining 14.0% of bands was eliminated during successive generations. Such an increase in the number of eliminated bands from F1 to F5 is quite surprising, as it would seem that most changes should take place directly after the allopolyploidization. Thus, according to the current study results, the greatest increase in genomic changes may be expected in the F5 generation or in the next generations, followed by a gradual decrease in the rate of transformations (Ma and Gustafson 2006). Similar trends were observed in newly synthesized *Brassica napus* allopolyploids (Lukens et al. 2006; Gaeta et al. 2007), in which genetic changes were very small in the first generation, but their frequency increased significantly to the F5 generation. Thus, research conducted in this study could theoretically concern the generations with the highest transformation intensity.

In conclusion, it can be argued that the significant elimination of DNA from the genome is the main process accompanying triticale evolution (Ma and Gustafson 2008). At the genome level, polyploids tend to drift towards diploid state by reducing the size of the genome and eliminating sequences, including duplicate genes (Wolfe 2001; Chen et al. 2007). Most of the studies conducted in allopolyploids confirmed that the DNA content tended to decrease compared to parental species. Therefore, the genomes of naturally occurring allopolyploids were smaller than they would be after the addition of parental genomes (Ozkan et al. 2003). Perhaps only partial elimination of certain sequences/chromosomes may be required to ensure the stability of the new allopolyploid species. The loss or gain of genomic DNA may be part of the speciation process, and the changes are species-specific, showing a directional or random nature.

### *Author contribution statement*

AK conceived, designed and performed the experiment; AK and MA designed and wrote the manuscript. Both authors read and approved the manuscript.

## Abbreviations

ISSR: Inter-simple sequence repeats
IRAP: Inter-retrotransposon amplified polymorphism
REMAP: Retrotransposon microsatellite amplified polymorphism
LMUR: Primary octoploidal triticale from Lanca × Muro cross
